# Conjugation of a Toll‐Like Receptor Agonist to Glycans of an HIV Native‐Like Envelope Trimer Preserves Neutralization Epitopes

**DOI:** 10.1002/cbic.202200236

**Published:** 2022-06-20

**Authors:** Zeshi Li, Ronald Derking, Wen‐Hsin Lee, Gerlof P. Bosman, Andrew B. Ward, Rogier W. Sanders, Geert‐Jan Boons

**Affiliations:** ^1^ Department of Chemical Biology and Drug Discovery Utrecht Institute for Pharmaceutical Sciences Utrecht University 3584 CG Utrecht The Netherlands; ^2^ Department of Medical Microbiology Amsterdam Institute for Infection and Immunity Amsterdam UMC University of Amsterdam 1105AZ Amsterdam The Netherlands; ^3^ Department of Integrative Structural and Computational Biology The Scripps Research Institute La Jolla CA, 92037 USA; ^4^ Department of Microbiology and Immunology Weill Medical College of Cornell University New York NY 10021 USA; ^5^ Complex Carbohydrate Research Center University of Georgia Athens GA 30602 USA; ^6^ Bijvoet Center for Biomolecular Research Utrecht University Utrecht The Netherlands; ^7^ Chemistry Department University of Georgia Athens GA 30602 USA

**Keywords:** carbohydrates, glycosyl transferases, conjugation, Toll-like receptor agonist, vaccines

## Abstract

Small molecule adjuvants are attractive for enhancing broad protection and durability of immune responses elicited by subunit vaccines. Covalent attachment of an adjuvant to an immunogen is particularly attractive because it simultaneously delivers both entities to antigen presenting cells resulting in more efficient immune activation. There is, however, a lack of methods to conjugate small molecule immune potentiators to viral glycoprotein immunogens without compromising epitope integrity. We describe herein a one‐step enzymatic conjugation approach for the covalent attachment of small molecule adjuvants to *N*‐linked glycans of viral glycoproteins. It involves the attachment of an immune potentiator to CMP‐Neu5AcN_3_ by Cu(I)‐catalyzed azide‐alkyne 1,3‐cycloaddition followed by sialyltransferase‐mediated transfer to *N*‐glycans of a viral glycoprotein. The method was employed to modify a native‐like HIV envelope trimer with a Toll‐like receptor 7/8 agonist. The modification did not compromise Env‐trimer recognition by several broadly neutralization antibodies. Electron microscopy confirmed structural integrity of the modified immunogen.

## Graphical abstract

**Viral glycoprotein immunogens** can be modified by a small molecule adjuvant by chemical attachment of an immune‐potentiator to CMP‐Neu5AcN_3_ followed by transfer to *N*‐glycans of the glycoprotein. The method was employed to modify a native‐like HIV envelop trimer with an agonist of Toll‐like receptor 7/8. The modification did not compromise structural integrity and Env‐trimer recognition by several broadly neutralization antibodies.

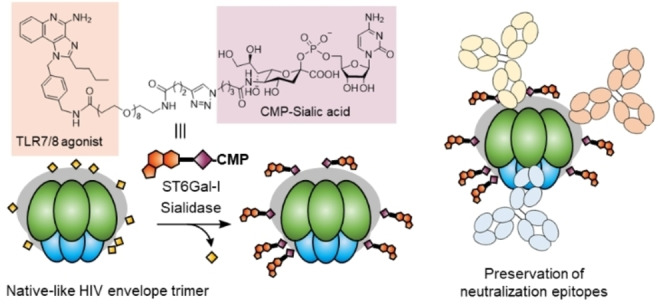

## Introduction

Subunit vaccines, in which only one or few microbial component(s) are administered, have greatly contributed to vaccine safety. For example, microbial toxins generated through recombinant DNA technology and altered to reduce toxicity, are successfully used as vaccines for a number of pathogens. In addition, polysaccharide‐protein conjugate vaccines have been developed as vaccines for *Haemophilus influenzae*, *Neisseria meningitidis* and *Streptococcus pneumoniae*.^[1]^

Although the subunit approach has many attractive features, it comes at the expense of decreased immunogenicity. This limitation is particularly problematic for immuno‐compromised patients and the elderly who often suffer from immuno‐senescence leading to decreased immune response to vaccination. In addition, it has been difficult to develop vaccines for diseases such as human immunodeficiency virus infection and acquired immunodeficiency syndrome (HIV/AIDS), malaria, *Mycobacterium tuberculosis*, *Plasmodium falciparum*, and hepatitis C virus (HCV). There are no licensed vaccines for nosocomial bacterial infections, such as *Pseudomonas aeruginosa*, *E. coli* and *Staphylococcus aureus*. It is clear new vaccine paradigms are needed to allow for more potently activating the immune system.

Tremendous progress has been made in understanding the molecular mechanisms that control immune cell activation.^[2]^ For example, receptors of the innate immune system have been identified that can recognize pathogen‐associated molecular patterns (PAMPs), which are generally conserved components of pathogens, such as nucleic acids, cell wall components, and flagellin that are not produced by humans. Recognition of PAMPs leads to activation of the innate immune system, which in addition to providing early protection, also shapes adaptive immune responses. This insight has led to the rationale development of adjuvants that can potentiate immune responses induced by subunit vaccines.^[3]^

The conjugation of PAMPs to an antigen of interest provides an attractive means to increase vaccine potency.^[4]^ In this approach, antigen and immune‐potentiator are delivered to the same antigen presenting cell resulting in more efficient immune activation. It makes it possible to reduce the adjuvant and antigen dose, thereby minimizing the risk of adverse effects. We and others pioneered this approach and demonstrated that a glycosylated cell surface associated mucin 1 (MUC1)‐derived glycopeptide covalently linked to a Toll‐like receptor (TLR) agonist can elicit potent humoral and cellular immune responses and is efficacious in reversing tolerance and generating a therapeutic response.^[5]^ Synthetic approaches make it possible to attach various small molecule adjuvants such as TLR2 and TLR7/8 agonists to synthetic antigens.^[4]^ Furthermore, recombinant DNA technologies have been used to make fusion proteins of antigen and a protein‐based immune‐potentiator such as flagellin,^[6]^ which is a TLR5 agonist, and HSP70^[7]^ and type‐III repeat extra domain A from fibronectin (EDA),^[8]^ which are TLR4 ligands.

Small molecule TLR agonists are a fast‐growing class of immune potentiators that are being explored as vaccine adjuvants,^[9]^ and robust methods are needed to attach such compounds to (glyco)protein antigens without compromising protein and antigenic integrity. UV cross‐linking has been employed to link a TLR7/8 agonist (TLR7/8a) to the HIV‐1 gag protein, which resulted in improved T‐cell immunity.^[10]^ In another study, a TLR7 ligand was attached to the model protein carrier, mouse serum albumin through a 2‐step procedure involving NHS activation of the protein followed by hydrazone mediated ligation.^[11]^ The resulting conjugate induced more potent *in vitro* and *in vivo* cytokine production. A TLR9 agonist (CpG) was attached to nitrophenol‐modified chicken gamma globulin as a model immunogen, which led to a higher number of follicular T helper cells in germinal centers and improved class‐switching, affinity maturation and memory responses.^[12]^ In the study above, nitrophenol‐modified chicken gamma globulin was biotinylated with biotin‐sulfosuccinimidyl ester which was mixed with biotin‐CpG1826 and then conjugated to streptavidin.

Although these studies have demonstrated the attractiveness of attaching a small molecule agonist to a protein antigen, the employed conjugation approaches are not suitable for many complex glycoprotein antigens. In this respect, conventional protein modification often exploits the nucleophilicity of lysine, arginine or cysteine side chains, and modification of these residues can compromise the integrity of important epitopes. For example, it has been shown that a two‐step conjugation of a TLR7/8a to HIV‐1 gp120 *via* lysine/arginine led to substantially decreased binding by broadly neutralizing antibodies (bNAbs).^[13]^ Furthermore, these conjugation approaches often involve non‐physiological pH and/or use of organic solvents, which can cause incompatibilities with sensitive antigens.

Herein, we describe a one‐step enzymatic conjugation approach for the covalent attachment of small molecule immune activators to asparagine‐linked glycans of viral glycoprotein immunogens. It involves pre‐attachment of a small molecule TLR7/8a to an azido‐modified β‐linked cytidine‐5’‐monophosphoryl sialic acid (CMP‐Neu5AcN_3_) derivative, which can then be transferred to *N*‐glycans of a viral glycoprotein by human sialyltransferase ST6Gal‐I. We demonstrate that the method can modify a fully‐glycosylated, native‐like HIV envelope (Env) trimer without loss of recognition by bNAbs.

**Figure 1:**
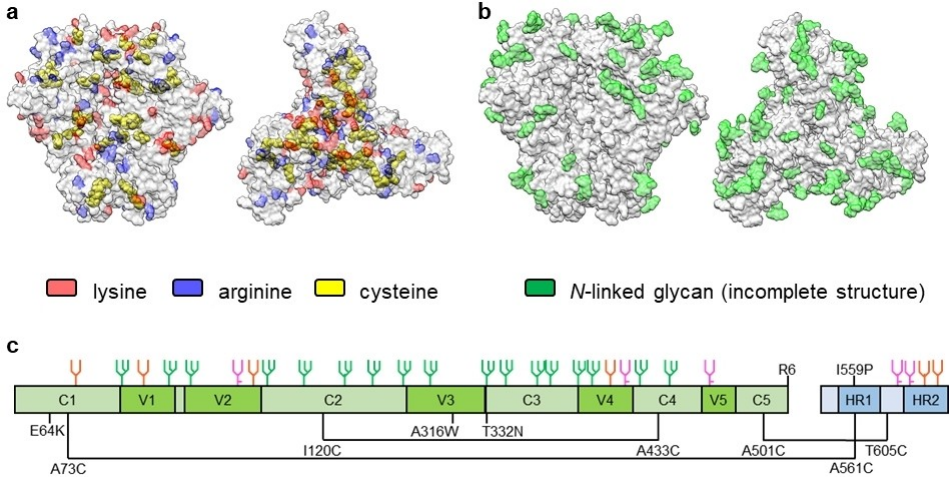
Potential sites for modification on a native‐like HIV envelope trimer. a) Lysine (red), arginine (blue) and cysteine (yellow) residues highlighted in the SOSIP native‐like Env trimer (PDB 6 V0R). Side view is on the left and top view is on the right. Protein surface is shown at 70 % transparency for cysteine residues (spherical) to be visible. Glycan structures are omitted. b) *N*‐linked glycans (incomplete structure, green, surface representation) present on the same protein. c) Linear schematic of native‐like Env trimer BG505 SOSIP v5.2^[14]^ was used in this study. Gp120 is shaded in green and gp41 in blue. Solid lines linking two cysteine residues indicate disulfide bonds. Glycan sites are color‐coded based on the glycoforms identified in site‐specific glycomic analysis.^[15]^ Sites shown in green are dominated by oligomannose‐type glycans (100–80 % oligomannose), in purple by hybrid‐/complex‐type glycans (39–0 % oligomannose); the sites where both types are present in similar abundance (79–40 % oligomannose) are shown in orange.

Mature native HIV‐1 Env spike proteins form a trimer of heterodimers (gp120 and gp41), which is rather unstable and readily dissociates. The intact trimeric state of the spike is critical for presenting neutralizing epitopes and reducing the exposure of non‐neutralizing epitopes which may lead to non‐relevant immune responses.^[16]^ An attractive strategy to stabilize the hexameric state of HIV Env spike, which is termed “SOSIP” (Figure 1c), is based on introducing several inter‐subunit disulfide bonds between Cys^501^ and Cys^605^, an Ile^559^Pro mutation to stabilize the protein complex in a prefusion state, and an improved furin (R6) cleavage site to facilitate maturation.[^16^, ^17^] Furthermore, additional mutations and disulfide bonds can be introduced to further stabilize the complex.^[14]^ SOSIP trimers are attractive vaccine candidates and we aimed to modify such a glycoprotein with a TLR7/8 agonist.

The HIV‐1 Env trimer contains up to 90 *N*‐linked glycans making up greater than half of its molecular weight (Figure 1b and c). The *N*‐glycan sites of the gp120 portion carries largely under‐processed oligomannosides, whereas the gp41 portion harbors a higher fraction of Golgi‐processed complex‐ and hybrid‐type *N*‐glycans. We envision that the sialic acid residues at the termini of the latter two glycan types may serve as attachment sites for desired functionalities. These glycan termini are at a relatively large distance from the protein surface, and their modification was expected to only marginally effect Env recognition by most bNAbs. An increasing number of bNAbs against multiple viral variants have been discovered which are valuable for guiding vaccine design.^[18]^ However, induction of such antibodies *via* immunization for broad and durable protection has not been realized yet.

**Figure 2:**
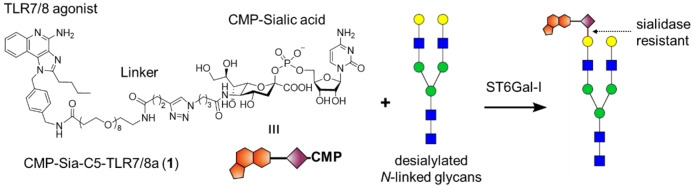
Strategy for protein modification. The conjugating agent **1** is composed of three parts: (1) a TLR7/8 agonist, (2) an oligo(ethylene glycol) spacer to increase aqueous solubility, and (3) a CMP‐activated carrier sialic acid to be transferred by ST6Gal‐I. Once attached to an N‐glycan, the glycosidic bond is insensitive to sialidase degradation.

Previously, we demonstrated that the human sialyltransferase, ST6Gal‐I, tolerates modifications at the C5‐postion of CMP‐Neu5Ac derivatives.^[19]^ Thus, it was anticipated that CMP‐Neu5Ac derivative **1** modified at C‐5 with a TLR7/8a can be transferred by ST6Gal1 to N‐glycans having terminal galactosyl residues. Furthermore, we have observed that a sialoside modified at C5 is resistant to *Clostridium perfringens* sialidase treatment. Thus, removal of endogenous sialic acid present on a glycoprotein and the installation of a C5‐modified counterpart can be performed as a one‐pot procedure (Figure 2).

## Results and Discussion

**Figure 3:**
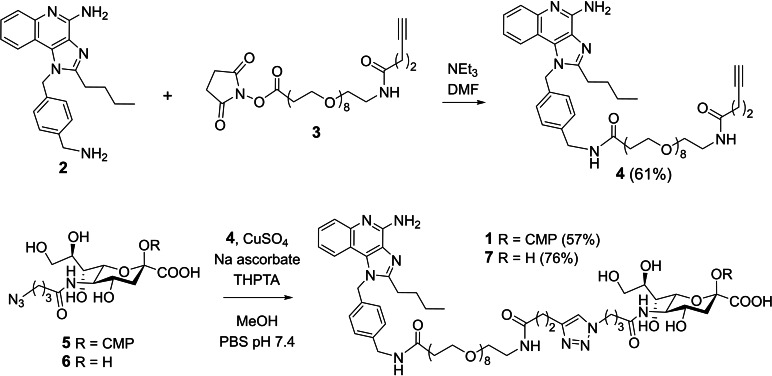
Preparation of conjugating agent 1 and non‐CMP‐activated **7**. Abbreviations: NEt_3_, triethylamine; DMF, *N*,*N*‐dimethylformamide; THPTA, tris(3‐hydroxypropyltriazolylmethyl)amine; MeOH, methanol; PBS, phosphate buffer saline.

CMP‐Neu5Ac derivative **1** was prepared by copper‐catalyzed click chemistry of the alkyne modified TLR7/8a **4** with CMP‐Neu5Ac derivative **5** having an azido‐moiety at C‐5 (Figure 3). Thus, the TLR7/8a‐alkyne portion (**4**), was prepared by acylation of the benzylic amine of TLR7/8a agonist **2** with *N*‐hydroxysuccinimide‐activated, oligo(ethylene glycol)‐spaced 4‐pentynoyl moiety **3**. The oligo‐ethylene glycol spacer was incorporated to increase water solubility. It has been reported that modification of the TLR7/8a **2** at the benzylic amine position with various functionalities does not compromise activity,^[20]^ making this drug molecule an appropriate candidate for bioconjugation. The TLR7/8a‐alkyne **4** was attached to CMP‐Neu5AcN_3_ (**5**) and free Neu5AcN_3_ (**6**) using *in situ* generated Cu(I) from CuSO_4_ and sodium ascorbate, and THPTA as an aqueously soluble ligand to give **1** and **7**, respectively.^[21]^ The CMP‐Sia‐linked TLR/8a **1** is completely water soluble, which eliminates the need for organic co‐solvent at the glycoprotein conjugation step. We also attempted to prepare compound **1** by condensation of **7** with CTP in the presence of *N. meningitidis* CMP‐Sia synthetase. However, the later enzyme does not tolerate large modifications at C‐5 of Neu5Ac and no product formation was observed.

**Figure 4:**
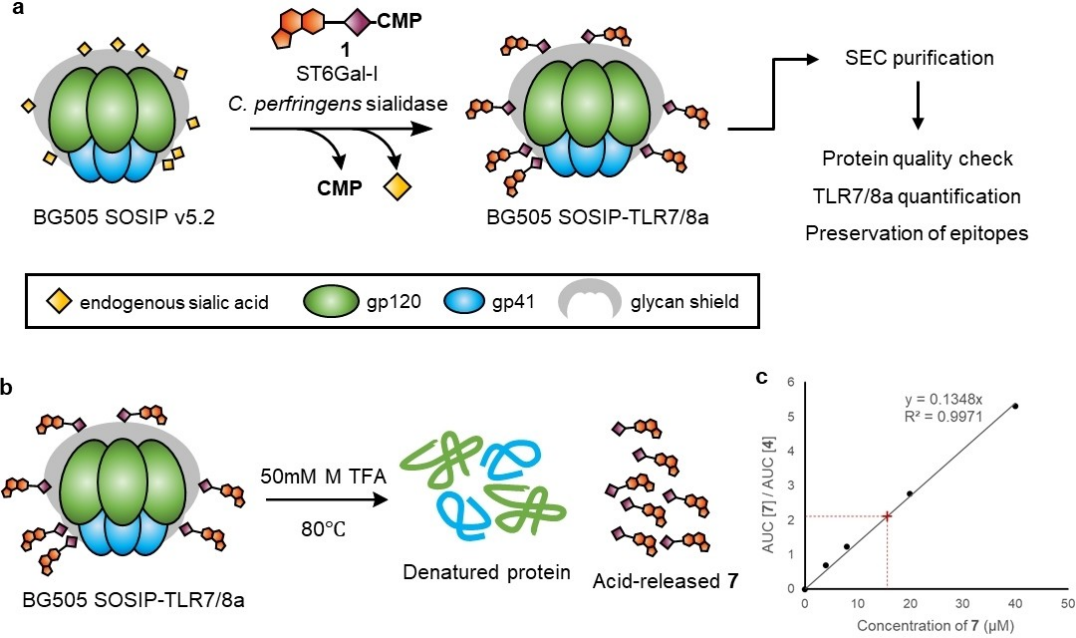
SOSIP‐trimer‐drug conjugation and characterization. a) One‐step de‐sialylation and re‐sialylation using sialyltransferase ST6Gal‐I and sialidase from *C. perfringens*. Post‐modification mixture was purified by size‐exclusion chromatography (SEC, see Supporting Figure S1 for chromatogram). An aliquot of the purified protein was taken for BCA analysis to determine the concentration. b) Reference‐free 2D class averages of negative stain electron microscopy images of purified SOSIP‐TLR7/8a. A total of 11,134 particles were analyzed, all of which are in the native‐like trimer form. c) Trifluoroacetic acid (TFA)‐mediated sialic acid cleavage for quantification. Released sialic acid‐TLR7/8a was subjected HPLC‐UV‐MS analysis. d) Standard curve of released sialic acid‐TLR7/8a species **7**. See Supporting Information for LC chromatograms of compound **7** at different concentrations. The red cross sign on the fitted trendline indicates observed normalized (against **4**) area under curve (AUC) values for sialic acid‐TLR7/8a released from BG505 SOSIP‐TLR7/8a protein.

Next, attention was focused on the modification of the native‐like BG505 SOSIP v5.2 Env trimer^[14]^ with compound **1**. Thus, the trimer was incubated with **1** in the presence of the sialidase from *C. perfringens* and ST6Gal‐I for 18 h. The trimer has a substantially larger molecular weight compared to ST6Gal1 and sialidase, and as a result could readily be purified by Superdex 200 size‐exclusion chromatography to give pure chimeric SOSIP trimer‐TLR7/8a conjugate (SOSIP‐TLR7/8a) as determined by the chromatogram and native gel electrophoresis (Figure S1a and S1b). The conjugated protein carrying TLR7/8a could not be quantified by nanodrop due to interference of UV absorption by the drug. Instead, a bicinchoninic acid (BCA) assay was used to determine the protein concentration (Figure S1c). Native gel electrophoresis, which showed SOSIP‐TLR7/8a remained intact post‐modification, does not have the resolving power to differentiate the TLR7/8a‐conjugated and non‐modified protein, due to the relatively small difference in molecular weight. To confirm successful modification, the SOSIP‐TLR7/8a conjugate and the non‐modified counterpart was treated with 50 mM trifluoroacetic acid (TFA) at 80 °C to release sialic acids (Figure 4c). Control experiments with pure water heated at the same temperature were also included. The resulting mixtures were subjected to high performance liquid chromatography coupled with a UV detector and a mass spectrometry (HPLC‐UV‐MS). Only TFA‐treated SOSIP‐TLR7/8a gave a signal in the chromatogram with a maximum absorption at 320 nm, and with a mass of the corresponding HPLC peak matching exactly that of chemically synthesized sialic acid‐TLR7/8a **7** (Figure S2).

To determine TLR7/8a‐to‐Env‐trimer ratio, we used chemically synthesized compound **7**, which has the same structure as the TFA‐cleaved sialic acid‐TLR7/8a species, to make a standard curve. Solutions of **7** in water at varying concentrations (40 to 4 μM) were prepared and subjected to HPLC‐UV analysis. Compound **4** was used at a concentration of 5 μM as an internal standard, because its retention time on HPLC differs considerably from **7**, yet having the same maximum absorption wavelength at 320 nm. The standard curve was obtained by plotting the normalized peak area of compound **7** in the chromatogram against its concentration, which made it possible to get the average number of the attached drug molecules after TFA‐mediated sialic acid release from SOSIP‐TLR7/8a (Figure 4d). The average attachment number was 13 (see Supporting Figure S3 for calculation), which falls well within the theoretical numbers of total complex *N*‐glycan sites on an Env trimer (see Supporting Information for calculation).

**Figure 5:**
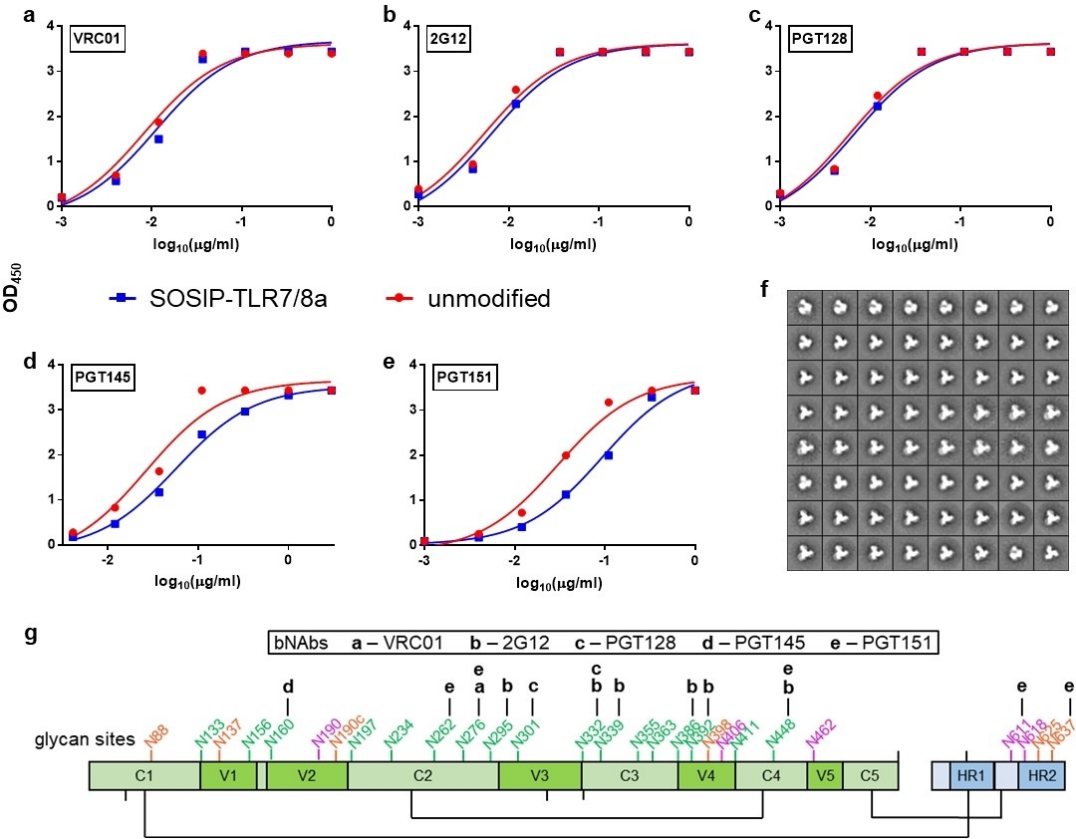
Glycan conjugation preserves neutralization epitopes. a–e) ELISA using the antigenic surface coated with TLR7/8a‐modified or native HIV Env trimer, probed with monoclonal antibodies VRC01 (a), 2G12 (b), PGT128 (c), PGT145(d) and PGT151 (e). f) NS‐EM 2D reference‐free class averages for TLR7/8a‐conjugated Env trimer. All 11,134 particles were found to be native‐like trimers. g) Linear schematic of SOSIP Env trimer showing glycan‐bNAb interactions. Glycans involved in binding are labeled with corresponding bNAb names. Glycan sites are color‐coded in the same fashion as Figure 1c. Amino acid labels are hidden for clarity (see Figure 1c).

Next, ELISA was used to examine if the protein modification preserves critical neutralization epitopes on the Env trimer (Figure 5). Thus, the TLR7/8a‐conjugate and non‐modified trimers carrying His‐tag were immobilized on Ni‐NTA‐coated plate. Primary bNAbs were added following a blocking step. bNAbs targeting multiple classes of neutralization epitopes, including ones that bind glycans were investigated and binding was detected using a goat‐anti‐human secondary antibody conjugated with HRP. As shown in panel **a**, the CD4 binding site bNAb VRC01^[22]^ was not affected by the modification. 2G12^[23]^ (panel **b**) and PGT128^[20b]^ (panel **c**) recognize Asn^332^‐centered high‐mannose glycans. The former binds glycans exclusively, whereas the latter also interacts with the V3 loop in addition to high‐mannose glycans. Neither of the bNAbs were affected by TLR7/8a conjugation. In the cases of trimer apex bNAb PGT145^[24]^ (panel **d**) and gp120–gp41 interface bNAb PGT151^[25]^ (panel **e**), potency was decreased by a modest 2–3 folds, indicating the modification was well‐tolerated by these bNAbs.

It is important that modification of a complex immunogen, such as HIV Env trimer, preserves its structural integrity. We employed negative stain electron microscopy (NS‐EM) to characterize the conjugated Env trimer. It was found that after TLR7/8a conjugation, the Env trimers had remained native‐like without detectable malformed trimers (Figure 5f). This is consistent with the binding of PGT145 which requires a trimeric state of Env. Collectively, the results indicate that the method can be employed to modify highly complex glycoprotein immunogens without compromising the integrity of neutralization epitopes.

Co‐administration of a TLR7 agonist with HIV envelope immunogens can induce more durable humoral responses.^[26]^ There is also data to support that the modification of a viral protein, such as HIV‐1 Env trimers, with a TLR agonist can increase immunogenicity.^[13]^ The challenge has, however, been to establish a conjugation methodology that allows the introduction of an immune‐potentiator without compromising the integrity of the glycoprotein and underlying bNAb epitopes. Here, we describe a mild and efficient one‐step procedure to modify glycoprotein immunogens, such as HIV‐1 Env trimers, with a small molecule adjuvant. Unlike lysine or cysteine‐based covalent attachment, the modification occurs at termini of glycans, which are well‐spaced away from the protein surface. bNAbs against multiple important neutralization epitopes were not or minimally affected by the modification, which indicates promise as a vaccine candidate, which will be the focus of future studies.

Previously, gp120 was modified by an unnatural monosaccharide by metabolic labeling and click chemistry technology for imaging purposes.^[27]^ It resulted in glycoproteins having low labeling efficiency ranges from 8–17 %. Remarkably, the one‐step modification using exogenously administered ST6Gal‐I achieved an average incorporation of 43 % per complex *N*‐glycosylation site (see Supporting Information for calculation).

The slight reduction in binding of trimer apex‐specific bNAb PGT145 and gp120–gp41 interface targeting bNAb PGT151 is in concordance with previous reports. These two bNAbs make contacts with a mixed population of *N*‐glycans. Specifically, PGT145 interacts asymmetrically with the N‐glycans at Asn^160^ of two gp120 subunits and exhibits a preference for oligomannose type glycan, whereas complex type glycans including α2,6‐sialylated glycoform has a negative impact on recognition due to possible steric clashes.[^24b^, ^28^] Asn^160^ indeed harbors a small fraction of Golgi‐processed complex‐type *N*‐glycans, which can be sialylated.[^15a^, ^15b^] PGT151 does prefer tri‐ and tetra‐antennary complex type glycans present on gp41.[^25^, ^29^] α2,6‐Sialylation has been reported to somewhat affect antibody binding of the epitope,^[28]^ which is consistent with our observations. Thus, the small reduction binding is probably caused by an altered glycoform on the Env protein upon sialylation.

Future studies will focus on the evaluation of immunogenicity of the conjugated Env trimer for their ability to activate innate immune cells such as dendritic cells and macrophages, as well as to induce neutralizing antibodies against HIVs. We expect the approach can be extended to other viral glycoprotein immunogens for vaccine development, such as for influenza A virus hemagglutinin and SARS‐CoV‐2 spike protein, both harboring a considerable number of complex/hybrid type *N*‐glycans sites for conjugation.^[30]^ In case the sialyl transferase and target viral glycoprotein have similar molecular weights, unique tags in the sialyl transferase make separation possible.

## Conclusions

A one‐step procedure has been developed to modify a native‐like HIV envelope trimer with a small molecule TLR7/8 agonist. It entails attachment of an immune potentiator to CMP‐Neu5AcN_3_ by Cu(I)‐catalyzed azide‐alkyne 1,3‐cycloaddition followed by sialyltransferase‐mediated transfer to the *N*‐glycans of the viral glycoprotein. The drug‐to‐protein ratio could readily be quantified by cleaving the modified sialic acid from the modified protein and subjecting the resulting sample to chromatographic analysis. A large range of broadly neutralizing monoclonal antibodies recognized the modified glycoprotein indicating it has promise for vaccine development.

## Experimental Section

Experimental details can be found in the Supporting Information.

## Conflict of interest

R.W.S. is an inventor on patents dealing with stabilized native‐like HIV‐1 envelope trimers.

1

## Supporting information

As a service to our authors and readers, this journal provides supporting information supplied by the authors. Such materials are peer reviewed and may be re‐organized for online delivery, but are not copy‐edited or typeset. Technical support issues arising from supporting information (other than missing files) should be addressed to the authors.

Supporting Information

## Data Availability

The data that support the findings of this study are available in the supplementary material of this article.
