# Supplementary material for: Conjugation of a Toll‐Like Receptor Agonist to Glycans of an HIV Native‐Like Envelope Trimer Preserves Neutralization Epitopes
Source: Chembiochem. 2022 Jun 20;23(16):e202200236. doi: 10.1002/cbic.202200236 (PMC9510654; doi:10.1002/cbic.202200236)
Supplement: Supplementary file 1 — Supporting Information [file CBIC-23-0-s001.pdf]

ChemBioChem

Supporting Information

Conjugation of a Toll-Like Receptor Agonist to Glycans of an HIV Native-Like Envelope Trimer Preserves Neutralization Epitopes

Zeshi Li, Ronald Derking, Wen-Hsin Lee, Gerlof P. Bosman, Andrew B. Ward, Rogier W. Sanders, and Geert-Jan Boons*

Table of Contents

Supplementary Figures and Text **2**

Experimental Section **6**

References **12**

Copies of NMR Spectra **13**

Supplementary Figures and Text

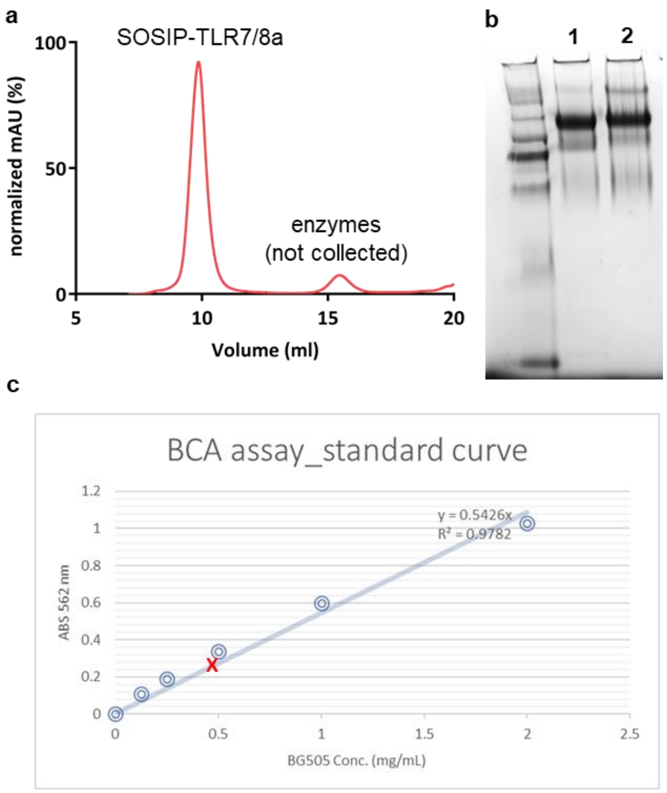

Supplementary Figure S1 | Protein purification and quality control. a, Size exclusion chromatogram (SEC) of TLR7/8a-conjugated Env trimer. SEC was performed on the Superdex Increase 200, eluting with 20 mM Tris/HCl 75mM NaCl pH 8. **b**, Non-denaturing Native-PAGE of the HIV envelope trimer before (Lane No. **1**) and after (Lane No. **2**) conjugation.[1] **c**, BCA assay using unmodified native-like HIV envelope trimer BG505 SOSIP v5.2 to plot the standard curve. The red cross sign indicates the ABS value obtained for the stock solution of SEC-purified modified trimer SOSIP-TLR7/8a. The ABS_{562} value for the solution is 0.26 (baseline subtracted). Concentration of the stock solution is therefore 0.48 mg/mL.

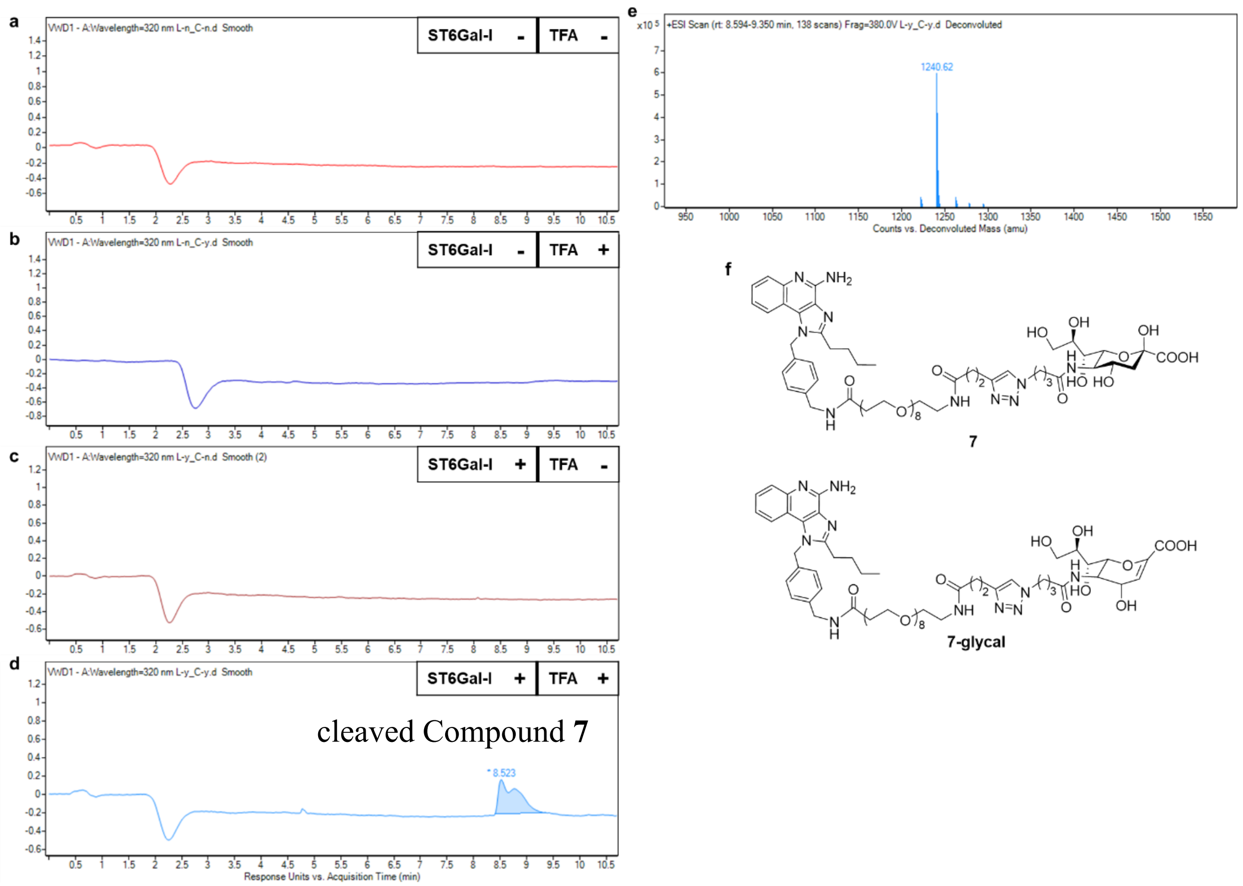

Supplementary Figure S2 | Confirmation of TLR7/8 agonist conjugation using chromatographic analysis. a-d, HPLC chromatograms of native-like HIV envelope trimer before or after modification, with (+) or without (-) 50 mM trifluoroacetic acid treatment. In **a** and **b**, the glycoprotein was incubated with compound **1** and sialidase but in the absence of sialyltransferase ST6Gal-I. In **a** and **c**, samples were heated at 80 °C in MilliQ water. 40 µg of protein was used for each group. Acquity UPLC peptide BEH C18 column was used, and eluted with a gradient of 0 – 50% acetonitrile at 0.3 mL/min flow rate within a period of 15 min. UV detector was set at 320 nm. **e**, Deconvoluted mass spectrum of the shouldered peak eluted around 8.5 min in panel **d** as a confirmation of presence of cleaved C5-TLR7/8a-conjugated sialic acid. Cleaved Compound **7** means the free sialic acid derivative released from the protein by TFA. Positive mode was used. **f**, There is a minor cleaved species with mass unit of 1222, which likely was given rise to by glycal (loss of water from free-reducing sialic acid). However, the abundancy of this peak is marginal compared with the major species and does not interfere with the quantification demonstrated in Fig. S3.

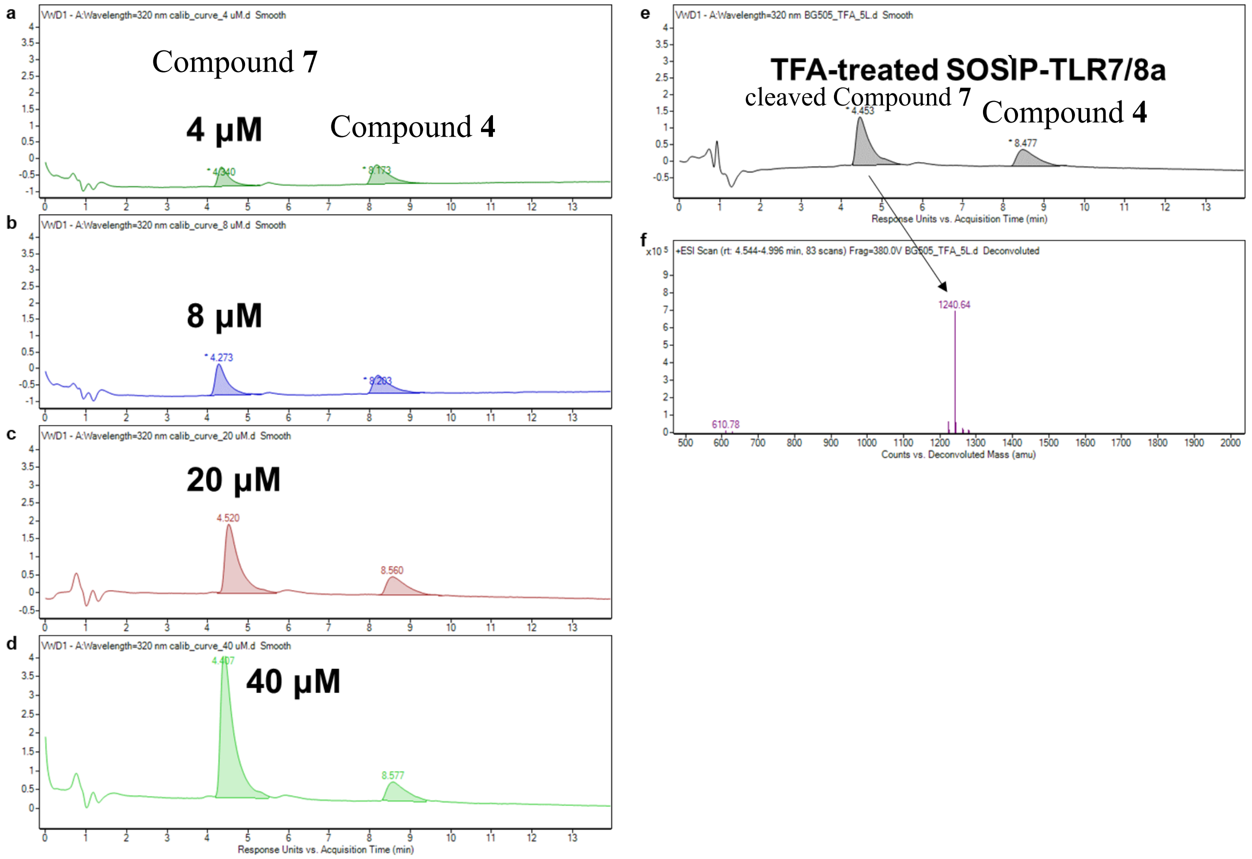

Supplementary Figure S3 | Preparation of standard curves for compound 7. a-d, Chemically synthesized **7** has exactly the same structure as does acid-cleaved TLR-7/8 agonist modified sialic acid. The former was dissolved at different concentrations (4-40 µM) in a MilliQ water solution containing 5 µM compound **4** as internal standard for normalization. These solutions were subjected to HPLC-UV analysis, using Acquity UPLC peptide BEH C18 column, eluted with a gradient of 20-32% acetonitrile in a period of 12 min. UV detector was set at 320 nm. Area under curve (AUC, curve smoothened) was integrated manually using the spectrum-processing software MassHunter Qualitative Navigator. These values were normalized via dividing the AUCs for 4-5 min peaks (compound **7**) by those for 8-9 min peaks (compound **4**). **e** and **f**, UV@320 nm chromatogram of 4.8 µg TFA-treated SOSIP-TLR7/8a and deconvoluted mass spectrum of 4-5 min peak, respectively. Cleaved Compound **7** means the free sialic acid derivative released from the protein by TFAPost-sialic acid release, the freeze-dried sample was reconstituted in 10 µL MilliQ containing the internal standard, a ratio of 2.1 (indicated with a red cross in Fig. 4c) was obtained for trifluoroacetic acid (TFA)-treated SOSIP-TLR7/8a, which gives a concentration of 15.6 µM. An estimated molecular weight of 400 kD was used for the protein, which gave a molarity of 1.2 µM. Thus, the average attachment number is 15.6/1.2 = 13.

Supplementary Text | Calculation of total complex *N*-glycosylation sites for a native-like HIV envelope trimer.

There are 24 N-glycan sites on a gp120 subunit and 4 on gp41. A native-like envelope trimer has hence a total of 84 N-glycan sites. The majority of glycan sites harbor a mixed population of glycoform types, which can be categorized into oligomannose/hybrid type (M/H) and complex type. The former is characterized by the sensitivity to degradation by glycosidase Endo H. Combining enzymatic treatment with sophisticated LC-MS/MS analysis, the M/H-type glycan content on a native-like envelope trimer has been determined, and has a value of 64%.[2] The rest 36% can be ascribed to complex type. Thus, the theoretical total number of complex glycan sites is 84*36% = 30. This means, on average, there are 30 glycans that could be potentially modified with the TLR7/8 agonist. Alternatively, a theoretical complex glycan sites can be calculated from an independent study,[3] where the fraction of complex glycans were provided for each glycan site. The sum value of the fraction (9.0) for a total of 28 sites is the theoretical complex glycan number on one copy of protomer. Hence the total number of complex glycan sites is 9*3 = 27, which is in agreement with the former calculated number.

Incorporation rate of the TLR7/8a into the protein in Discussion section was calculated by dividing the average attachment number (13) by the theoretical total number of complex glycan sites (30), presuming each glycan can only be modified with one drug due to the inherent N-glycan branch selectivity of ST6Gal-I. The labeling efficiency of metabolic glycan tagging mentioned earlier in the same paragraph, as reported,[4] was the ratio between the MS abundancy of an azide-tagged glycopeptide ions to the sum abundancy of the same glycopeptide with and without azide-tag.

Experimental Section

Materials and Methods

Unless otherwise stated, all reagents for chemical reactions were purchased from Merck Sigma-Aldrich. Solvents for reactions were taken from the solvent purifier (MB SPS 5) or purchased from Biosolve Chemie. Technical grade organic solvents in work-ups were from VWR Chemicals. Deuterated solvents for NMR experiments were purchased from Cambridge Isotope Laboratories. ST6Gal-I and were expressed and purified according to reported protocols.[5] Sialidase from *C. perfringens* was purchased from Merck Sigma-Aldrich (11585886001). CTP and CMP-Sia synthetase NmCSS for were purchased from Chemily, LLC. RP-HPLC analysis was performed using the column Acquity UPLC peptide BEH C18 column (300Å, 1.7 µm, 1 mm × 50 mm) from Waters. NMR spectra were collected on an Agilent 400-MR, VARIAN or Bruker 600 UltraShield. Brucker micrOTOF-Q II ESI mass spectrometer were used for MS-based reaction monitoring. Agilent 6560 ion mobility Q-ToF MS couple with HPLC system was used for drug attachment quantification and HRMS data. Enzymatic reactions are conducted using MaxQ 4450 incubator (Thermo Scientific) at 37 °C with shaking or in a water bath set at 37 °C. ELISA: Ni-NTA HisSorbTM plates (Qiagen 35061), 1-Step™ Turbo TMB-ELISA Substrate Solution (ThermoFisher Scientific, 34022). Non-fat milk (Biorad, 1706404). Native-PAGE: NuPAGE 4-12% Bis-Tris Gel (Novex NP0321BOX), 20x NativePAGE Running Buffer (Invitrogen BN2001), 20x NativePAGE Cathode Buffer Additive (Invitrogen BN2002), Coomassie Brilliant Blue G-250 (Fluka 27815), 100% Ultrapure Glycerol (Invitrogen 15514-011), HMW-Native Protein Mixture (GE Healthcare 17-0445-01). Negative stain electron microscopy (NS-EM) characterization was performed as previously described.[6]

Protein expression. BG505 SOSIP v5.2 were expressed and purified as previously described.[6] Briefly, HEK293T cells were cultured in Dulbecco's modified Eagle's medium (DMEM) containing 10% fetal calf serum (FCS), penicillin (100 U/mL), streptomycin (100 µg/mL), Glutamax (Invitrogen), non-essential amino acids (0.1 mM), sodium pyruvate (0.1 mM) and HEPES (0.1 mM). For gp140 trimer production, HEK293T cells were seeded at a density of 5.5×10^4/mL in a Corning Hyperflask. The cells reached a density of 1.0×10^6/mL after 3 days. To transfect the cells, PEI-MAX (1.0 mg/mL) in water was mixed with expression plasmids for Env and furin in OPTI-MEM. For one Corning Hyperflask, 600 µg of Env plasmid, 150 µg of Furin plasmid and 3 mg of PEI-MAX were added in 550 mL growth media. Culture supernatants were harvested 72 hours after transfection. The transfection supernatants were vacuum filtered through 0.2-µm filters and then passed (0.5–1 mL/min flow rate) over a PGT145 Sepharose column. The eluted proteins were buffer exchanged into 75 mM NaCl, 10 mM Tris, pH 8.0, using Snakeskin dialysis tubing (10K MWCO, Thermo Scientific). The proteins after dialysis were concentrated using Vivaspin columns with a 30-kDa cut off (GE Healthcare). The concentrated Env proteins were further purified using size exclusion chromatography (SEC) on a Superdex 200 26/60 column (GE Healthcare). The trimer fractions were collected and pooled. For NS-EM, BG505 SOSIP v8 was used.

Negative-stain electron microscopy. Trimer protein was diluted to 0.02mg/ml in 1x Tris-buffered saline, 3 μL applied to a 400mesh Cu grid, blotted with filter paper, and stained with 2% uranyl formate. Micrographs were collected at 52,000 magnification on a ThermoFisher Tecnai Spirit microscope operating at 120kV with a TVIPS TemCam F416 CMOS camera using Leginon automated image collection software.[7] Particles were picked using DogPicker[8] and 2D classification was done using iterative multivariate statistical analysis (MSA)/ multireference alignment (MRA).[9]

Protein structures are visualized in UCSF Chimera. Chemical structures were drawn using ChemDraw Professional 16.0. Diagrams were drawn with Microsoft PowerPoint. NMR spectra were processed and analyzed using MestReNova. ELISA results were graphed using Graphpad Prism 7. Standard curves were plotted using Microsoft Excel.

General Procedures for Protein Modification and Quantification

For chromatographic analysis demonstrated in Fig. S2, 40-50 μg protein was used, whereas a normal scale reaction is performed with 1-2 mg protein. 50 μg ST6Gal-I per mg of BG505 SOSIP v5.2, 0.1 U/mg sialidase from C. perfringens, 500 μM compound **1** were used. Enzymatic reaction was performed at a working concentration of 1-2 mg/mL BG505 SOSIP v5.2, and were placed in a 37 ℃ incubator or water bath for 16-20 hours.

For small scale modification, the solution was directly transferred to a 500 μL spin filter with a molecular weight cut-off (MWCO) of 100 kD. The upper vial was topped up to the maximum volume. The spin filter was centrifuged at 6000 rpm for 20 min (if the volume in the upper vial does not go below 50 μL, higher speed or longer time should be used) at 4 ℃ for seven times to ensure excess CMP-Sia derivative **1** was removed to the extent below detection limit. The spin-concentrated solution (25-50 μL) was transferred to a new vial for TFA-mediated sialic acid cleavage.

For normal scale, the labeling solution was subjected to size exclusion chromatography (Superdex Increase 200, elute with 20 mM Tris/HCl 75mM NaCl pH 8) for purification. The fractions were combined and concentrated using the spin filter (100 kD MWCO). Small aliquots were taken for BCA assay (Pierce™ BCA Protein Assay Kit 23227) and TFA treatment and LC-UV-MS attachment number determination. The aliquot (~5 μg) was diluted to ~100 μL with MilliQ and an equivolume of 100 mM TFA was added. The solution was heated at 80 ℃ for 1 hour and then dried with speed-vac. The residue was constituted with 10 μL of MilliQ which contains 5 μM compound **4** as internal standard and was injected in one goal into Acquity UPLC peptide BEH C18 column (20-32%). The peaks corresponding to **1** (~4.5 min) and **4** (~8.5 min) were integrated manually. The same chromatographic condition was applied to the chemically synthesized **7** at different concentrations (4, 8, 20, 40 μM) for making the standard curve.

Compound Numbering for NMR Assignments

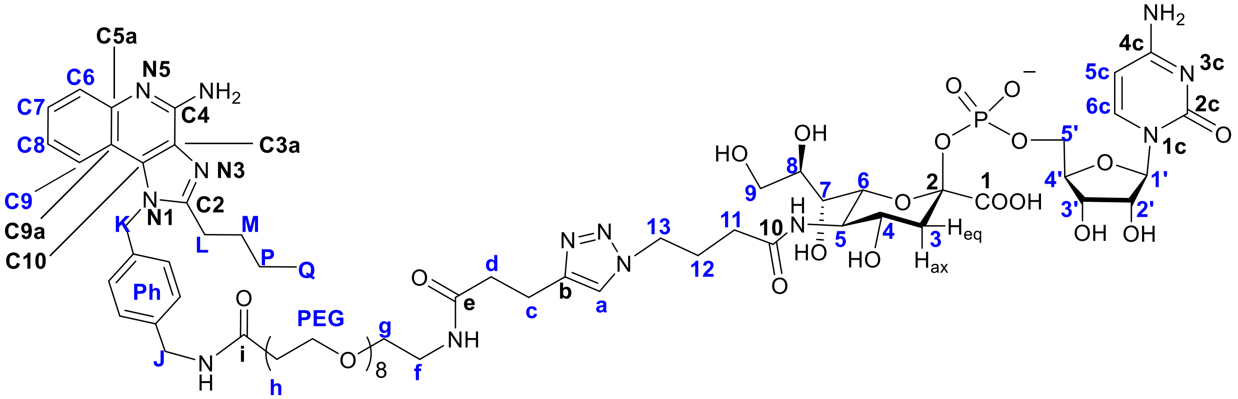

Numbering was shown using compound **1** and the rest of the compounds follow the same numbering scheme. In compound **4**, position a is an alkyne instead of a double bond in trizole moiety. The signals for positions shown in blue are assigned.

Detailed synthetic procedures

Synthesis of compound 4

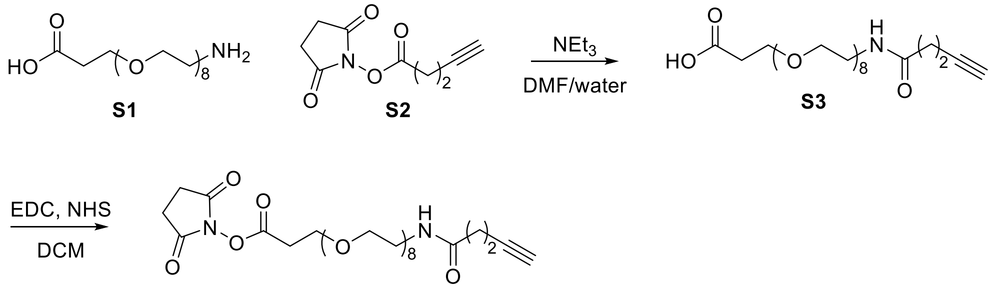

3 (64% over 2 steps)

TLR7/8a as benzylamine derivative **2** was prepared following reported procedures.[10] Alkyne-containing linker **3** was prepared from carboxylic acid-PEG8-amine **S1**. Specifically, in a round bottom flask, the starting material (330 mg, 0.74 mmol) was dissolved in 5 mL DMF/water (4:1). To this solution was added triethylamine (2 eq., 1.5 mmol, 0.2 mL) and *N*-hydrosuccinimide-activated 4-pentynoic acid **S2** (1.3 eq., 0.96 mmol, 200 mg). The reaction was stirred at 21 °C for 1 hour. The solution was concentrated in vacuo, and co-evaporated with toluene for 3 times. The residue was loaded on a LH-20 column to remove excess reagents. The acylated linker S3 was taken up in (8 mL) DCM, to which N-hydroxysuccinimide (NHS, 2 eq. to **S1**, 1.5 mmol, 169 mg) was added. N-(3-Dimethylaminopropyl)-N′-ethylcarbodiimide (EDC)·HCl was added at 0 °C. The reaction was then allowed to warm up to 21°C and stirred for 1 hour. The reaction was then diluted with DCM and washed ice cold 1N HCl solution and brine, and dried over sodium sulfate. After

filtration, the organic solution was concentrated in vacuo to give compound **3** as a crude. This was used directly to acylate compound **2** without further purification.

Compound **2** as TFA salt (13 mg, 28 µmol) was dissolved in 0.3 mL DMF in a 15 mL Falcon tube, to which triethylamine (3 eq., 0.84 µmol, 12 µL) and **3**, which was prepared as a 0.5 M solution in DMF, was added (1.5 eq. 42 µmol, 80 µL). The reaction was stirred at 21°C for 1 hour. DMF was removed in vacuo by speed-vac with heating at 45°C. The residue was directly subjected to preparative reverse phase HPLC purification using a C8 column (20-65% acetonitrile/water, 12 mL/min, 50 min). Of note, the crude compound was taken up in methanol for injection. 28 mg compound **4** was obtained in 61% isolated yield. See table below for ^{1}H- and ^{13}C-NMR assignment and HRMS.

Compound 4: ***N*-(4-((4-amino-2-butyl-1*H*-imidazo[4,5-c]quinolin-1-yl)methyl)benzyl)-1-(pent-4-ynamido)-3,6,9,12,15,18,21,24-octaoxaheptacosan-27-amide**					
MeOD-*d4,* 600 MHz					
	δH (ppm)	δC (ppm)		δH (ppm)	δC (ppm)
**C6**	7.77	117.5	**a**	2.28	68.49
**C7**	7.39	124.3	**c**	2.46	13.49
**C8**	7.66	128.8	**d**	2.4	33.82
**C9**	7.99	120.8	**f**	3.36	38.28
**K**	5.59	47.68	**g**	3.6-3.4[a]	69-70
**J**	4.38	41.32	**h**	2.46	35.59
**L**	3.02	25.66	**Q**	0.96	12.00
**M**	1.87	28.18	**Ph**	7.32	127.2
**P**	1.48	21.23		7.05	124.6
HRMS (ESI positive mode), calculated for $C_{46}H_{67}N_6O_{10}$ $[M+H]^+$ 863.4919, found 863.4929.					
[a] Peak buried in the PEG signal region.					

Synthesis of compounds 1 and 7

Compound **4** (12 mg, 14 µmol) was dissolved in 1 mL methanol, to which 1 mL aqueous solution of compound **5** (1.0 eq. 21 µmol, 15mg) or 1 mL methanol solution of **6** (1.5 eq. 21 µmol, 8 mg) was added. When water was added to the methanol containing **4**, the solution turned cloudy due to low water solubility of **4**. The following reagents for click reaction were pre-mixed in water as a solution: $CuSO_4$ (0.5 eq. 1.7 mg as $CuSO_4{\cdot}5H_2O$, 7 µmol), THPTA (0.8 eq. 4.9 mg, 11 µmol), sodium ascorbate (1.0 eq. 2.7 mg, 14 µmol). This solution has a volume of 0.5 mL, and was immediately added to the solutions containing **5** or **6**. Upon addition of the click reagents, the solution of **4** mixed with **5** rapidly turned from cloudy to clear. These two solutions were stirred at 21°C for an hour. The reactions were monitored using ESI-MS (negative mode for **4**+**5** and positive mode for **4**+**6**). Methanol was removed by blowing nitrogen gently to the solution. Then the light

blue water solutions were freeze-dried. The residue compounds were loaded onto biogel p2 for purification (elution: 50 mM ammonium bicarbonate, 4℃ for compound **1** and 21℃ for compound **7**). 12 mg compound **1** and 13 mg compound **7** were obtained. Isolated yields: 57 and 76%, respectively. See tables below for ^{1}H- and ^{13}C-NMR assignment and HRMS.

Compound 7: **5-(*N*-(4-((4-amino-2-butyl-1*H*-imidazo[4,5-c]quinolin-1-yl)methyl)benzyl)-1-(3-(1-(4-(methylamino)-4-oxobutyl)-1*H*-1,2,3-triazol-4-yl)propanamido)-3,6,9,12,15,18,21,24-octaoxaheptacosan-27-amido)-3,5-di-deoxy-D-glycero-D-galacto-2-nonulosonic acid**					
D_2O, 400 MHz					
	δH (ppm)	δC (ppm)		δH (ppm)	δC (ppm)
**C6**	7.61	119.6	**3eq**	2.20	39.53
**C7**	7.3	125.0	**3ax**	1.82	
**C8**	7.58	129.6	**4**	4.01	67.14
**C9**	7.76	121.0	**5**	3.93	52.2
**K**	5.73	48.48	**6**	3.97	70.13
**J**	4.32	42.34	**7**	3.61-3.26[a]	68-70
**L**	2.94	26.28	**8**	3.74	70.21
**M**	1.67	28.84	**9**	3.82	62.2
**P**	1.33	21.61			
**Q**	0.83	12.93	**11**	2.23	32.21
**Ph**	7.24	127.7	**12**	2.12	25.63
	6.97	125.6	**13**	4.34	49.21
**a**	7.71	121.2	**f**	3.28	38.8
**c**	2.89	20.98	**g**	3.61-3.26[a]	68-70
**d**	2.52	34.93	**h**	2.47	36.12
HRMS (ESI positive mode), calculated for $C_{59}H_{90}N_{10}O_{21}$ $[M+2H]^{2+}$ 1242.6383, found 621.3196 (z=2).					
[a] Peak buried in the PEG signal region.					

Compound 1: **Cytidine-5’-monophospho-5-(*N*-(4-((4-amino-2-butyl-1*H*-imidazo[4,5-c]quinolin-1-yl)methyl)benzyl)-1-(3-(1-(4-(methylamino)-4-oxobutyl)-1*H*-1,2,3-triazol-4-yl)propanamido)-3,6,9,12,15,18,21,24-octaoxaheptacosan-27-amido)-3,5-di-deoxy-D-glycero-D-galacto-2-nonulosonic acid**				
D_2O, 600 MHz				
	δH (ppm)	δC (ppm)	multiplet	*J* (Hz)
**C6**	7.63	120.6	d	9.0
**C7**	7.16	123.5	dd	7.6, 9.0
**C8**	7.49	128.4	dd	7.6, 9.0
**C9**	7.69	123.0	d	-[a]
**K**	5.61	48.28	s	
**J**	4.33	42.39	s	

**L**	2.89	26.36	t	-
**M**	1.62	29.23	m	-
**P**	1.33	21.76	m	-
**Q**	0.84	13.01	t	7.4
**Ph**	7.23	127.7	d	8.0
	6.93	125.8	d	8.0
**a**	7.61	123.7	s	
**c**	2.91	20.99	t	-
**d**	2.54	35.01	t	7.5
**f**	3.30	38.89	t	-
**g**	3.60-3.16[b]	68-70	-	-
**h**	2.48	36.25	t	5.5
**3eq**	2.52	41.33	dd	4.6, 12.0
**3ax**	1.67		d(t)[c]	12.0, -
**4**	4.09	66.85	ddd	4.6, 10.7, 11.1
**5**	3.99	51.82	dd	10.4, 11.1
**6**	4.16	71.78	dd	10.6, -
**7**	3.60-3.16[b]	68-70	-	-
**8**	3.96	69.63	ddd	2.2, 6.5, -
**9a**	3.88	62.97	dd	2.1, 12.0
**9b**	3.64		dd	6.4, 12.0
**11**	2.26	32.36	m	-
**12**	2.15	25.78	m	-
**13**	4.35	49.27	t	6.6
**5c**	6.06	96.47	d	7.6
**6c**	7.94	141.5	d	7.6
**1'**	5.93	89.03	d	4.3
**2'**	4.27	74.36	dd	-
**3'**	4.33	69.25	dd	-
**4'**	4.23	82.91	dt	-
**5'a, b**	4.25	64.84	t	-

HRMS (ESI negative mode), calculated for $C_{68}H_{98}N_{13}O_{26}$ $[M-2H]^{2-}$ 1543.6484, found 771.8248 (z=2).

[a] "-" Indicates coupling constant is not determined due to signal overlap.
[b] Peak buried in the PEG signal region.
[c] Axial H-3 of sialic acid becomes a dt peak upon CMP attachment.

References

[1] L. Cao, J. K. Diedrich, D. W. Kulp, M. Pauthner, L. He, S. R. Park, D. Sok, C. Y. Su, C. M. Delahunty, S. Menis, R. Andrabi, J. Guenaga, E. Georgeson, M. Kubitz, Y. Adachi, D. R. Burton, W. R. Schief, J. R. Yates, III, J. C. Paulson. *Nat. Commun.* **2017**, *8*, 14954.

[2] K. Sliepen, B. W. Han, I. Bontjer, P. Mooij, F. Garces, A. J. Behrens, K. Rantalainen, S. Kumar, A. Sarkar, P. J. M. Brouwer, Y. Hua, M. Tolazzi, E. Schermer, J. L. Torres, G. Ozorowski, P. van der Woude, A. T. de la Pena, M. J. van Breemen, J. M. Camacho-Sanchez, J. A. Burger, M. Medina-Ramirez, N. Gonzalez, J. Alcami, C. LaBranche, G. Scarlatti, M. J. van Gils, M. Crispin, D. C. Montefiori, A. B. Ward, G. Koopman, J. P. Moore, R. J. Shattock, W. M. Bogers, I. A. Wilson, R. W. Sanders. *Nat. Commun.* **2019**, *10*, 2355.

[3] A. J. Behrens, S. Vasiljevic, L. K. Pritchard, D. J. Harvey, R. S. Andev, S. A. Krumm, W. B. Struwe, A. Cupo, A. Kumar, N. Zitzmann, G. E. Seabright, H. B. Kramer, D. I. Spencer, L. Royle, J. H. Lee, P. J. Klasse, D. R. Burton, I. A. Wilson, A. B. Ward, R. W. Sanders, J. P. Moore, K. J. Doores, M. Crispin. *Cell Rep.* **2016**, *14*, 2695-2706.

[4] L. Sun, M. Ishihara, D. R. Middleton, M. Tiemeyer, F. Y. Avci. *J. Biol. Chem.* **2018**, *293*, 15178-15194.

[5] K. W. Moremen, A. Ramiah, M. Stuart, J. Steel, L. Meng, F. Forouhar, H. A. Moniz, G. Gahlay, Z. Gao, D. Chapla, S. Wang, J. Y. Yang, P. K. Prabhakar, R. Johnson, M. D. Rosa, C. Geisler, A. V. Nairn, J. Seetharaman, S. C. Wu, L. Tong, H. J. Gilbert, J. LaBaer, D. L. Jarvis. *Nat. Chem. Biol.* **2018**, *14*, 156-162.

[6] J. M. Binley, R. W. Sanders, B. Clas, N. Schuelke, A. Master, Y. Guo, F. Kajumo, D. J. Anselma, P. J. Maddon, W. C. Olson, J. P. Moore. *J. Virol.* **2000**, *74*, 627-643.

[7] C. Suloway, J. Pulokas, D. Fellmann, A. Cheng, F. Guerra, J. Quispe, S. Stagg, C. S. Potter, B. Carragher. *J. Struct. Biol.* **2005**, *151*, 41-60.

[8] N. R. Voss, C. K. Yoshioka, M. Radermacher, C. S. Potter, B. Carragher. *J. Struct. Biol.* **2009**, *166*, 205-213.

[9] T. Ogura, K. Iwasaki, C. Sato. *J. Struct. Biol.* **2003**, *143*, 185-200.

[10] N. M. Shukla, C. A. Mutz, R. Ukani, H. J. Warshakoon, D. S. Moore, S. A. David. *Bioorg. Med. Chem. Lett.* **2010**, *20*, 6384-6386.

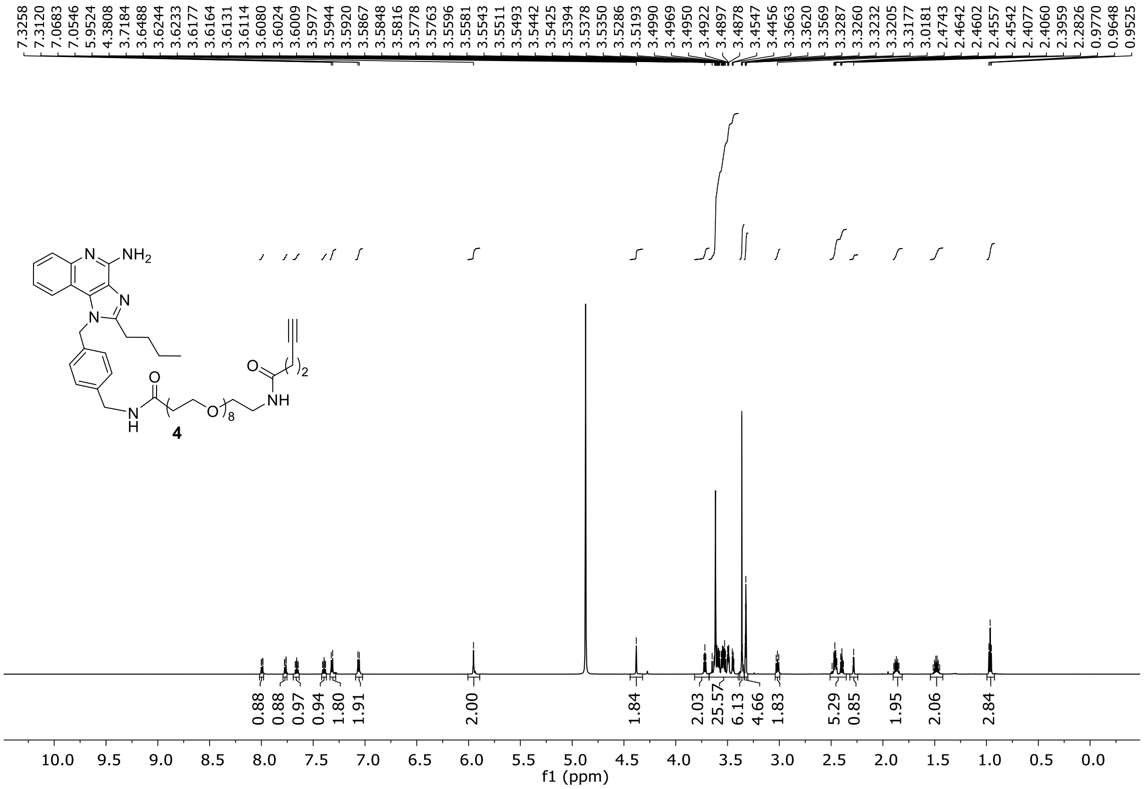
7.3258
7.3120
7.0683
7.0546
5.9524
4.3808
3.7184
3.6488
3.6244
3.6233
3.6177
3.6164
3.6131
3.6114
3.6080
3.6024
3.6009
3.5977
3.5944
3.5920
3.5867
3.5848
3.5816
3.5778
3.5763
3.5596
3.5581
3.5543
3.5511
3.5493
3.5442
3.5425
3.5394
3.5378
3.5350
3.5286
3.5193
3.4990
3.4969
3.4950
3.4922
3.4897
3.4878
3.4547
3.4456
3.3663
3.3620
3.3569
3.3287
3.3260
3.3232
3.3205
3.3177
3.0181
2.4743
2.4642
2.4602
2.4557
2.4542
2.4077
2.4060
2.3959
2.2826
0.9770
0.9648
0.9525
NH2
N
N
N
O
O
NH
N
H
O
8
2
4
0.88
0.88
0.97
0.94
1.80
1.91
2.00
1.84
2.03
25.57
6.13
4.66
1.83
5.29
0.85
1.95
2.06
2.84
10.0 9.5 9.0 8.5 8.0 7.5 7.0 6.5 6.0 5.5 5.0 4.5 4.0 3.5 3.0 2.5 2.0 1.5 1.0 0.5 0.0
f1 (ppm)

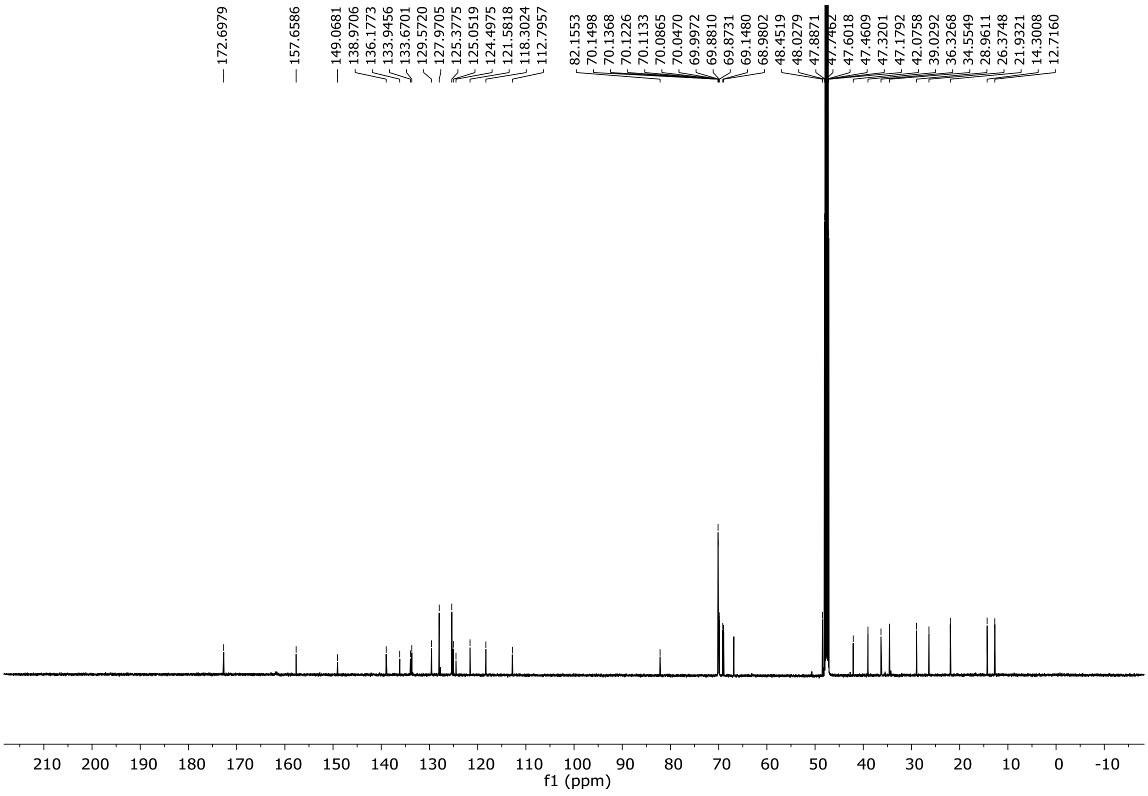
172.6979
157.6586
149.0681
138.9706
136.1773
133.9456
133.6701
129.5720
127.9705
125.3775
125.0519
124.4975
121.5818
118.3024
112.7957
82.1553
70.1498
70.1368
70.1226
70.1133
70.0865
70.0470
69.9972
69.8810
69.8731
69.1480
68.9802
48.4519
48.0279
47.8871
47.7462
47.6018
47.4609
47.3201
47.1792
42.0758
39.0292
36.3268
34.5549
28.9611
26.3748
21.9321
14.3008
12.7160
210 200 190 180 170 160 150 140 130 120 110 100 90 80 70 60 50 40 30 20 10 0 -10
f1 (ppm)

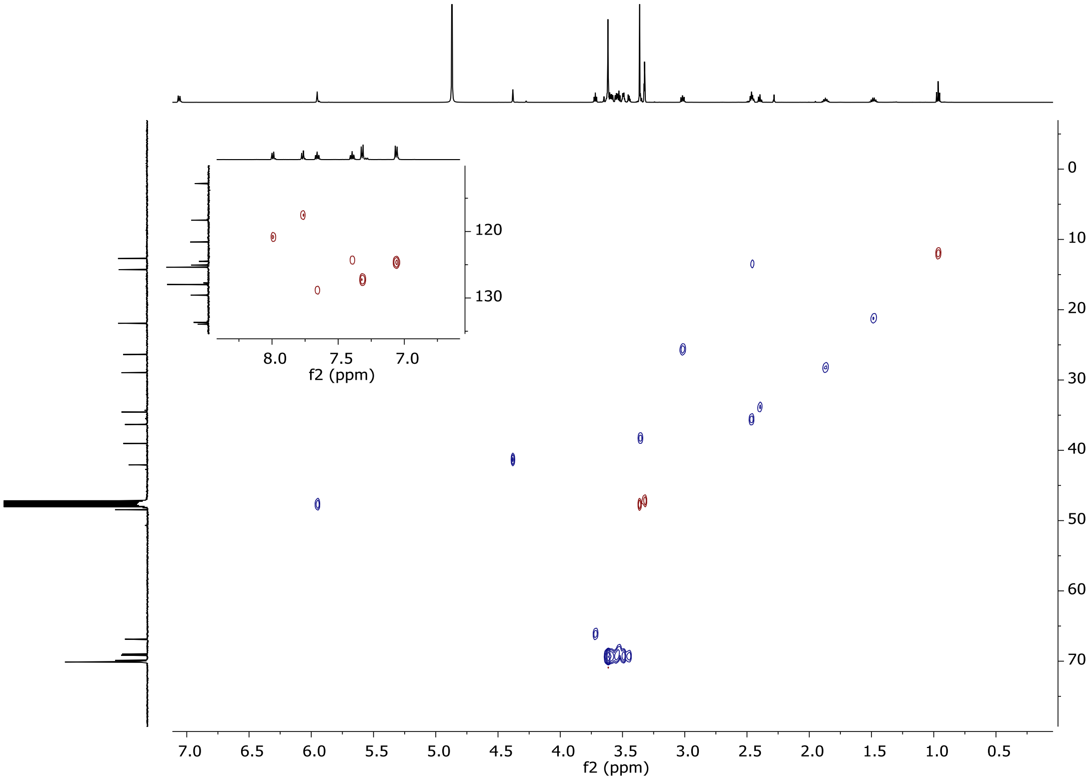
8.0
7.5
7.0
f2 (ppm)
120
130
0
10
20
30
40
50
60
70
7.0
6.5
6.0
5.5
5.0
4.5
4.0
3.5
3.0
2.5
2.0
1.5
1.0
0.5
f2 (ppm)

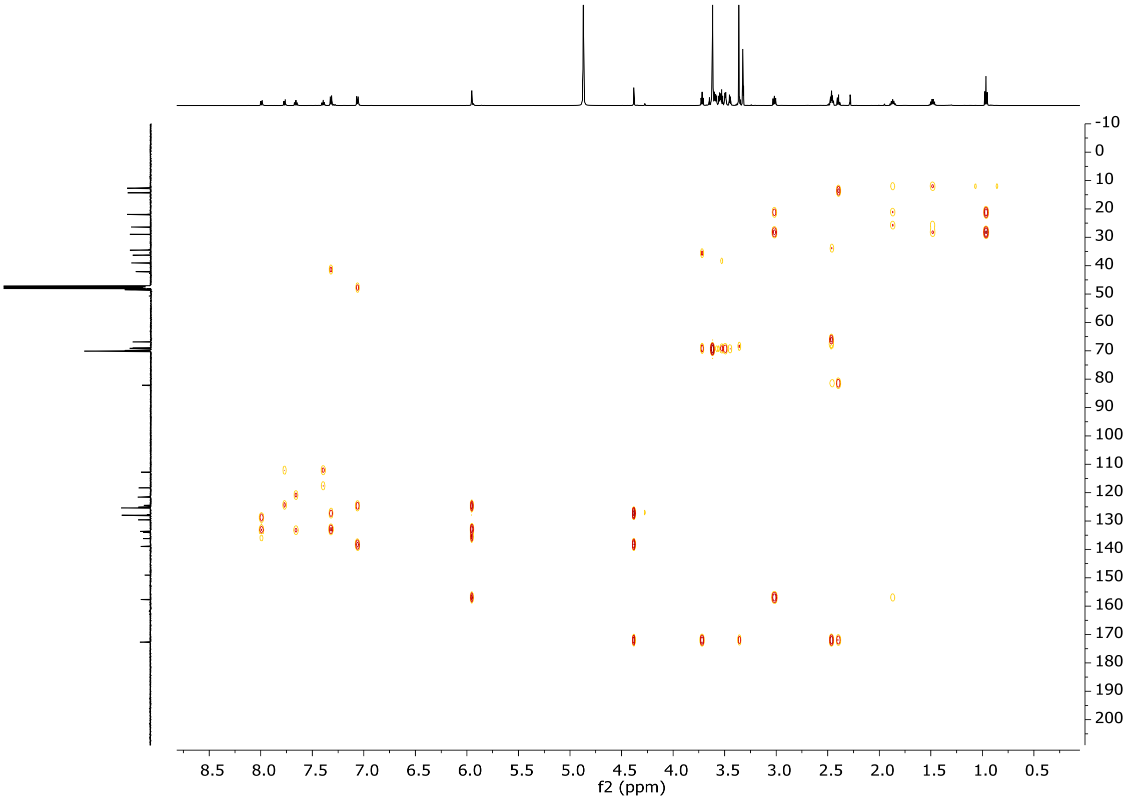
-10
0
10
20
30
40
50
60
70
80
90
100
110
120
130
140
150
160
170
180
190
200
8.5
8.0
7.5
7.0
6.5
6.0
5.5
5.0
4.5
4.0
3.5
3.0
2.5
2.0
1.5
1.0
0.5
f2 (ppm)

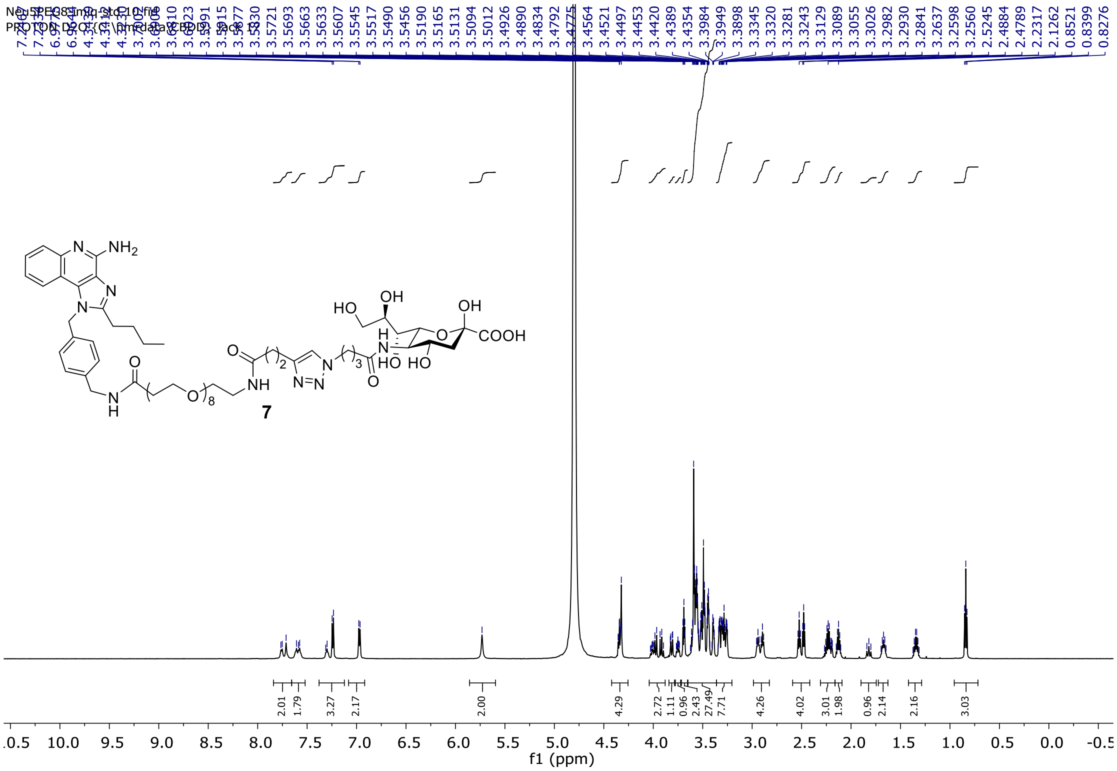
NH2
HO
OH
OH
COOH
HO
HO
NH
7
f1 (ppm)

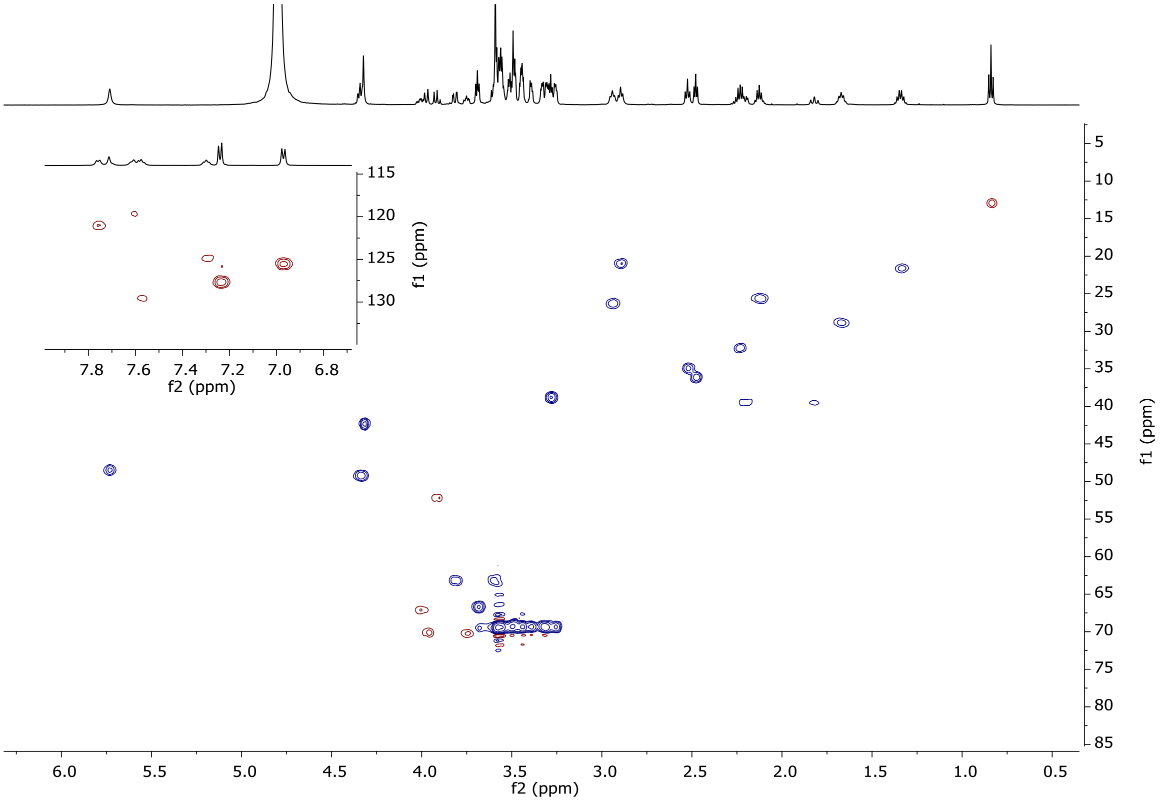
f2 (ppm)
f1 (ppm)
f2 (ppm)
f1 (ppm)

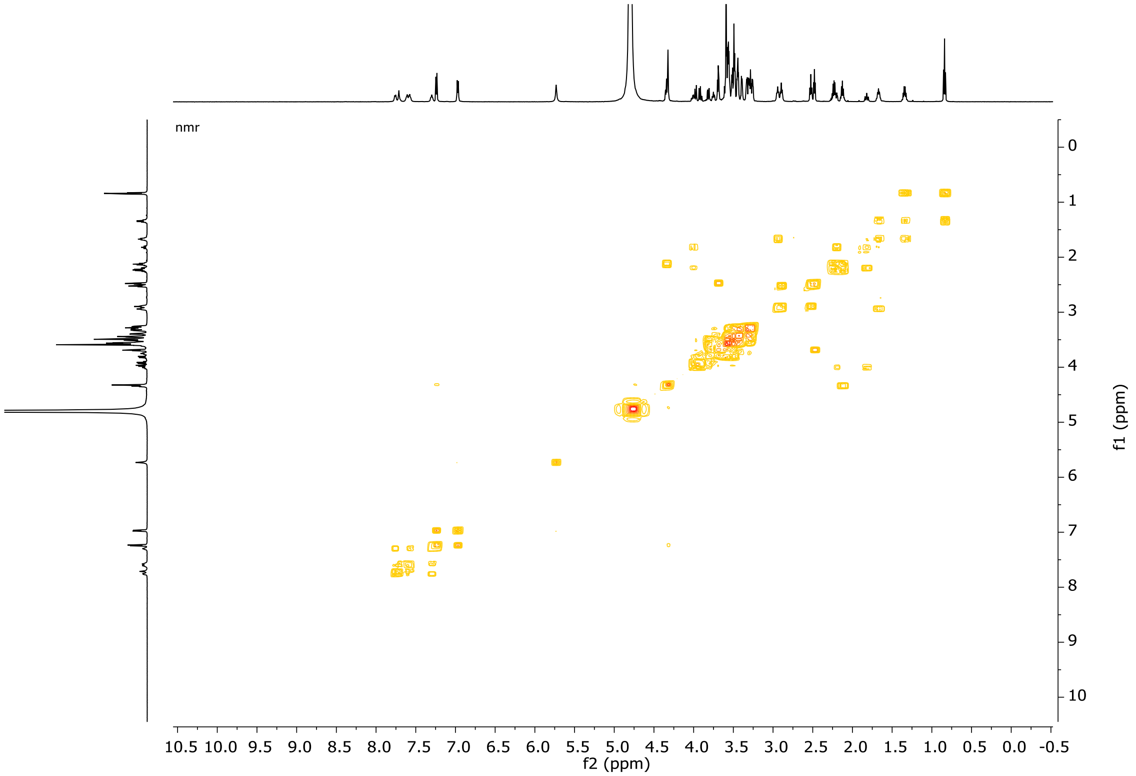
nmr
f1 (ppm)
f2 (ppm)

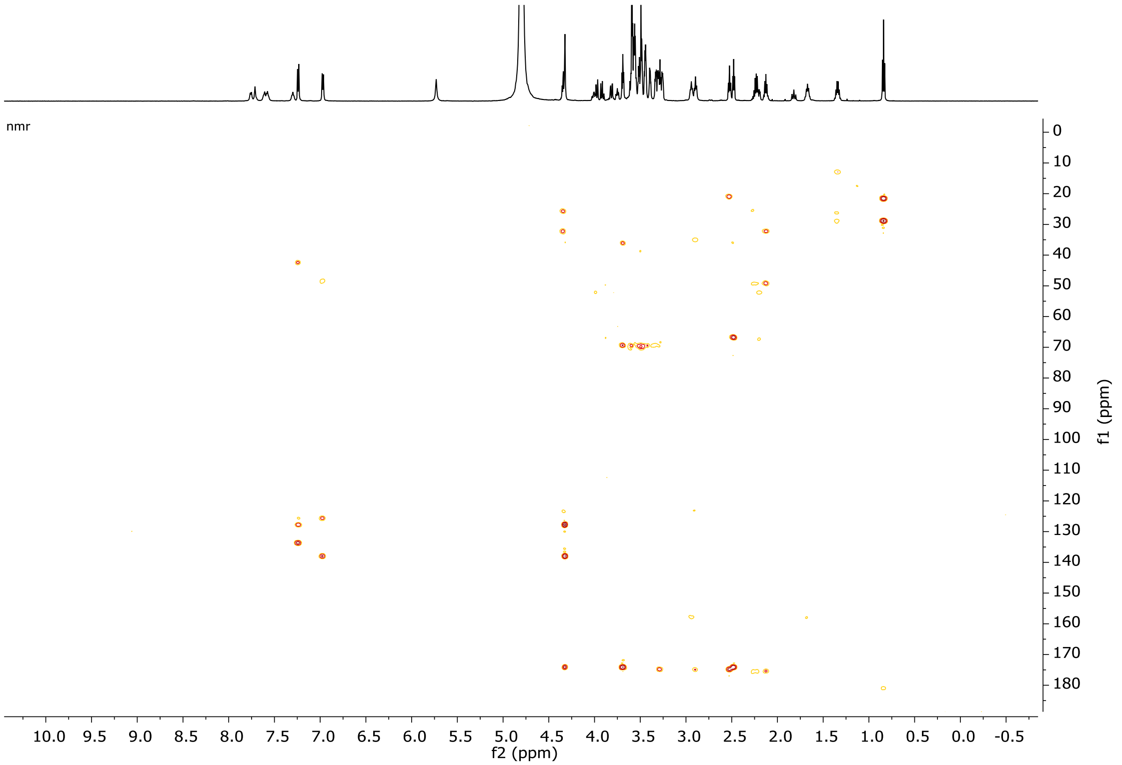
nmr
f1 (ppm)
f2 (ppm)

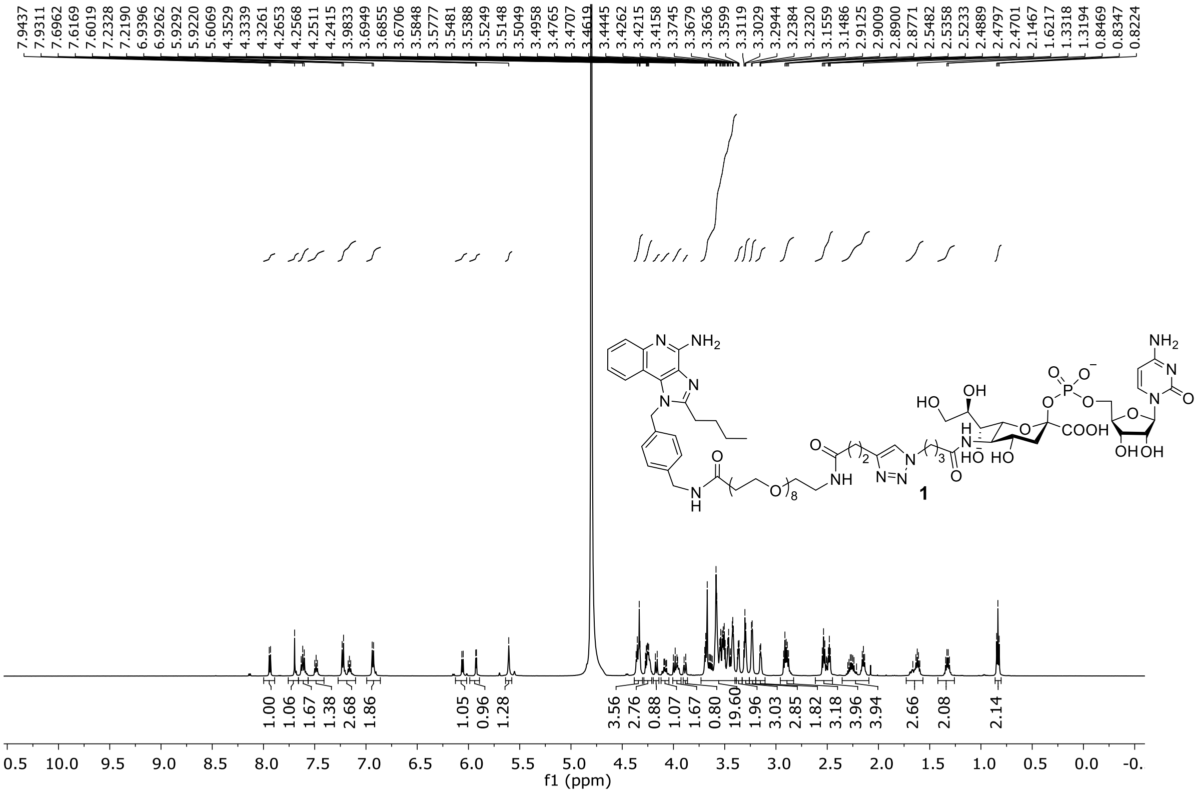
7.9437 7.9311 7.6962 7.6169 7.6019 7.2328 7.2190 6.9396 6.9262 5.9292 5.9220 5.6069 4.3529 4.3339 4.3261 4.2653 4.2568 4.2511 4.2415 3.9833 3.6949 3.6855 3.6706 3.5848 3.5777 3.5481 3.5388 3.5249 3.5148 3.5049 3.4958 3.4765 3.4707 3.4619 3.4445 3.4262 3.4215 3.4158 3.3745 3.3679 3.3636 3.3599 3.3119 3.3029 3.2944 3.2384 3.2320 3.1559 3.1486 2.9125 2.9009 2.8900 2.8771 2.5482 2.5358 2.5233 2.4889 2.4797 2.4701 2.1467 1.6217 1.3318 1.3194 0.8469 0.8347 0.8224
NH2
N
N
OH
HO
O
COOH
1
1.00 1.06 1.67 1.38 2.68 1.86 1.05 0.96 1.28 3.56 2.76 0.88 1.07 1.67 0.80 19.60 1.96 3.03 2.85 1.82 3.18 3.96 3.94 2.66 2.08 2.14
f1 (ppm)
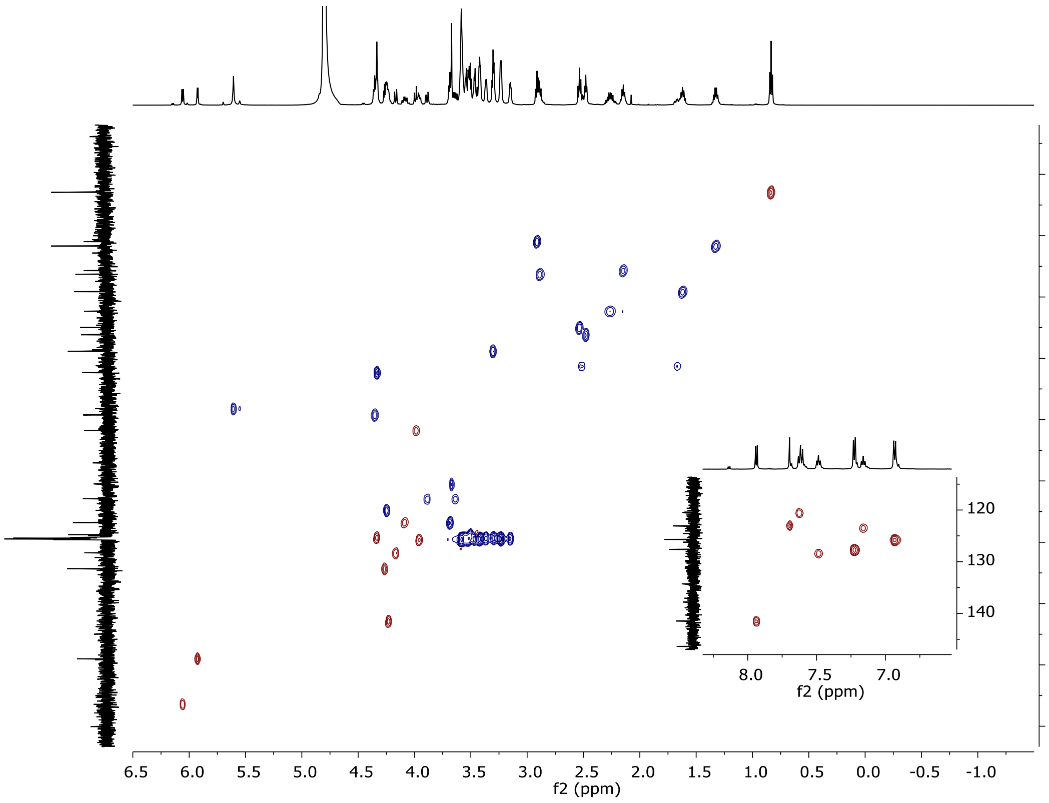
f2 (ppm)
